# Regulatory T Cells Fail to Suppress Fast Homeostatic Proliferation In Vitro

**DOI:** 10.3390/life11030245

**Published:** 2021-03-16

**Authors:** Daniil Shevyrev, Valeriy Tereshchenko, Elena Blinova, Nadezda Knauer, Ekaterina Pashkina, Alexey Sizikov, Vladimir Kozlov

**Affiliations:** 1 Laboratory of Clinical Immunopathology, Research Institute for Fundamental and Clinical Immunology, 630099 Novosibirsk, Russia; blinovaelena-85@yandex.ru (E.B.); knauern@mail.ru (N.K.); pashkina.e.a@yandex.ru (E.P.); vakoz40@yandex.ru (V.K.); 2 Laboratory of Molecular Immunology, Research Institute for Fundamental and Clinical Immunology, 630099 Novosibirsk, Russia; tervp@ngs.ru; 3 Rheumatology Department, Research Institute for Fundamental and Clinical Immunology, 630099 Novosibirsk, Russia; depaidici@online.nsk.su

**Keywords:** Treg, rheumatoid arthritis, homeostatic proliferation, Treg function, index of suppression

## Abstract

Homeostatic proliferation (HP) is a physiological process that reconstitutes the T cell pool after lymphopenia involving Interleukin-7 and 15 (IL-7 and IL-15), which are the key cytokines regulating the process. However, there is no evidence that these cytokines influence the function of regulatory T cells (Tregs). Since lymphopenia often accompanies autoimmune diseases, we decided to study the functional activity of Tregs stimulated by HP cytokines from patients with rheumatoid arthritis as compared with that of those from healthy donors. Since T cell receptor (TCR) signal strength determines the intensity of HP, we imitated slow HP using IL-7 or IL-15 and fast HP using a combination of IL-7 or IL-15 with anti-CD3 antibodies, cultivating Treg cells with peripheral blood mononuclear cells (PBMCs) at a 1:1 ratio. We used peripheral blood from 14 patients with rheumatoid arthritis and 18 healthy volunteers. We also used anti-CD3 and anti-CD3 + IL-2 stimulation as controls. The suppressive activity of Treg cells was evaluated in each case by the inhibition of the proliferation of CD4^+^ and CD8^+^ cells. The phenotype and proliferation of purified CD3^+^CD4^+^CD25^+^CD127^lo^ cells were assessed by flow cytometry. The suppressive activity of the total pool of Tregs did not differ between the rheumatoid arthritis and healthy donors; however, it significantly decreased in conditions close to fast HP when the influence of HP cytokines was accompanied by anti-CD3 stimulation. The Treg proliferation caused by HP cytokines was lower in the rheumatoid arthritis (RA) patients than in the healthy individuals. The revealed decrease in Treg suppressive activity could impact the TCR landscape during lymphopenia and lead to the proliferation of potentially self-reactive T cell clones that are able to receive relatively strong TCR signals. This may be another explanation as to why lymphopenia is associated with the development of autoimmune diseases. The revealed decrease in Treg proliferation under IL-7 and IL-15 exposure can lead to a delay in Treg pool reconstitution in patients with rheumatoid arthritis in the case of lymphopenia.

## 1. Introduction

During the course of a person’s life, the human body is frequently exposed to different stresses, infections, chemicals, radiation, and other physical agents that can potentially lead to the development of lymphopenia. It is well known that an increased risk of autoimmune diseases, including rheumatoid arthritis, is associated with lymphopenia, which can play a crucial role in the early stages of disease development [1]. The principal physiological mechanism for restoring the T cell pool after lymphopenia is homeostatic proliferation (HP) [2], which is the main source of T cells after the thymus’ involution in adulthood [3]. It is well known that the T cell receptor (TCR) signal, co-stimulation signals, and IL-7 and IL-15 are the main factors that facilitate homeostatic proliferation (HP) [4,5,6,7,8]. Various cell types produce IL-7 and IL-15, primarily in lymphoid tissues under lymphopenia [4,9,10,11]. These cytokines both belong to the common cytokine receptor γ-chain (γ_c_) family and signal through receptor complexes that contain the γ_c_ (IL-2Rγ) subunits CD127 and CD215, respectively. IL-7 and IL-15 typically activate the PI3K-Akt, RAS-RAF-MAPK, and JAK-STAT signaling pathways, which, together with TCR signaling, mediate the survival and proliferation of T lymphocytes during HP [12,13]. It should be noted that based on intensity, HP can be contingently divided into slow and fast. HP intensity directly depends on the TCR signal strength determined by the avidity of the TCR–pMHC interaction (pMHC, peptide in major histocompatibility complex) [14,15]. Weak or strong TCR signals generate different Akt proteoforms, which determine the activation of different intracellular signaling pathways and thus affect cell fate [16]. Therefore, the division of HP into slow and fast has a materialistic background.

For slow HP, the presence of a “tonic” TCR signal and increased levels of IL-7 and IL-15 are sufficient. It is a polyclonal proliferation for maintaining the diversity of the TCR repertoires and probably does not lead to negative changes in the immune system. By contrast, fast HP depends on a strong TCR signal, is oligoclonal, and changes the structure of the general TCR repertoire. During fast HP, T cells are formed with effector and memory-like phenotypes. Altogether, these changes during fast HP increase the risk of tissue inflammation and autoimmune disease development [17,18,19]. Since the avidity of the TCR–pMHC interaction determines the ability of T cells to compete for proliferation factors in lymphopenic conditions, only 30% of the total pool of T lymphocytes, characterized by the highest TCR–pMHC affinity, enters HP in vivo. Since not all possible antigens are present in the body simultaneously and the majority of antigens are self-antigens, potentially self-reactive T cell clones are selected during HP, changing the overall T cell landscape [6,18,19,20,21,22].

Tregs are the main cells providing tolerance to self-antigens through a wide range of mechanisms. It has been shown that Tregs are able to inhibit T cell proliferation and cytokine production, thus preventing autoimmunity [22]. However, the role of these cells in the suppression of self-reactive T cell clones during homeostatic proliferation is understudied. It is assumed that a decrease in Treg quantity and their impaired function may lead to the development of autoimmune diseases [23]. At the same time, the results from existing research on Treg cell function in patients with rheumatoid arthritis (RA) remain controversial. While some studies demonstrate decreases in Treg numbers or suppressive activity in RA patients [24,25,26,27,28], others show the preservation of their functional activity and quantity [29,30,31,32]. Nevertheless, despite the increased synovial infiltration of Tregs in RA patients [33], the inflammation does not resolve, raising the question as to whether their suppressive activity is actually preserved. Thus, this study was aimed at determining the functional activity of Tregs in RA patients and investigating the influence of humoral factors of HP on Tregs in vitro. For that purpose, we investigated the capacity of Tregs to inhibit the proliferation of CD4^+^ and CD8^+^ cells stimulated by various HP humoral factors. We used IL-7 or IL-15 alone and in combination with anti-CD3 (analogs of slow and fast HP, respectively). Since the TCR signal and IL-2 are the most important factors for the maintenance of Treg function, we also stimulated proliferation with an anti-CD3 + IL-2 combination. Thus, we used the following stimulation conditions: anti-CD3 only, anti-CD3 + IL-2, IL-7 only, anti-CD3 + IL-7, IL-15 only, anti-CD3 + IL-15, Tregs pretreated with IL-7 or IL-15 stimulated by anti-CD3, and a control without stimulation.

## 2. Materials and Methods

### 2.1. Participants

Eighteen healthy donors (HDs) and fourteen patients with RA (according to the ACR/EULAR 2010 criteria) were enrolled in this study conducted at the Immunopathology Hospital Rheumatology Department of the Research Institute for Fundamental and Clinical Immunology, Novosibirsk, Russia. The Local Ethics Committee, in accordance with the Declaration of Helsinki (minutes no. 110, 11 October 2018), approved the study. In all the cases, blood was sampled following written voluntary informed consent. The study included patients with a polyarticular form of rheumatoid arthritis with medium and high disease activity treated for disease exacerbation at the Research Institute Rheumatology Department. Disease activity was evaluated using the 28-joint disease activity score (DAS-28), which was, on average, 5.2 ± 1.6. The average duration of the disease was 5.5 ± 2.7 years (Table 1). All the patients received disease-modifying antirheumatic drugs (DMARDs), i.e., methotrexate or sulfasalazine as monotherapies or combined with glucocorticoids. The exclusion criteria included an acute or active infection, lactation or pregnancy, biological DMARD or targeted therapy application, and a history of cancer or immune deficiency, as well as other immune-related diseases (e.g., type I diabetes and chronic kidney or liver disease). There were no significant differences in sex or average age between the HDs and RA patients.

### 2.2. Cell Sorting and Treg Suppressive Activity Assay

Peripheral blood mononuclear cells (PBMCs) were isolated using Ficoll density gradient centrifugation. Treg cells were purified by immunomagnetic separation according to the CD3^+^CD4^+^CD25^+^CD127^low^ phenotype using a Miltenyi Biotec MACS Treg Isolation Kit, according to the manufacturer’s protocol (Teterow, Germany). The purity of the magnetic separation was 94.2 ± 3% (mean ± SD), and FoxP3 expression was checked by flow cytometry and was 83 ± 3.5% (mean ± SD), with no difference between the healthy donors and RA patients. The functional activity of the Tregs was evaluated by the inhibition of CD4^+^ and CD8^+^ lymphocyte proliferation with a Treg/PBMC autologous co-culture ratio of 1:1 (i.e., 30,000 Tregs and 30,000 PBMCs). Before cultivation, PBMCs depleted by Tregs were stained with CFSE (Invitrogen, Waltham, MA, USA) to assess proliferation. The cells were cultivated over 4 days in 96-well U-bottom plates with RPMI 1640 medium supplemented with 10% FCS and antibiotics, in a total volume of 200 µL. The following concentrations of cytokines were applied: anti-CD3 (Sorbent, Moscow, Russia), 1 µg/mL; IL-7 (MyBiosource, San Diego, CA, USA), 50 ng/mL; IL-15 (MyBiosource, San Diego, CA, USA), 50 ng/mL; IL-2 (NPK BIOTECH, St. Petersburg, Russia), 100 IU/mL. We used the following stimulation conditions: anti-CD3, IL-7, IL-15, anti-CD3 + IL-2, anti-CD3 + IL-7, and anti-CD3 + IL-15. To directly measure HP cytokines’ effects, Tregs were previously incubated with IL-7 (50 ng/mL) or with IL-15 (50 ng/mL) over 2 h, washed twice, and then cultivated with PBMCs stimulated by anti-CD3. CFSE-stained PBMCs were cultivated under the same conditions without Treg cells (60,000 per well) to calculate the suppression index (SI). Cells were also cultivated without any stimulation applied, as the cell proliferation control. The Treg suppression index (SI) was calculated for CD4^+^ and CD8^+^ cells using the following formula [34]:

(1) $$\mathrm{SI}=100\times \left(1-\frac{\;\%\;\mathrm{Tconv}\;\mathrm{proliferation}\;\mathrm{with}\;\mathrm{Treg}}{\%\;\mathrm{of}\;\mathrm{Tconv}\;\mathrm{proliferation}\;\mathrm{in}\;\mathrm{the}\;\mathrm{absence}\;\mathrm{of}\;\mathrm{Treg}}\right)$$

**Figure 1:**
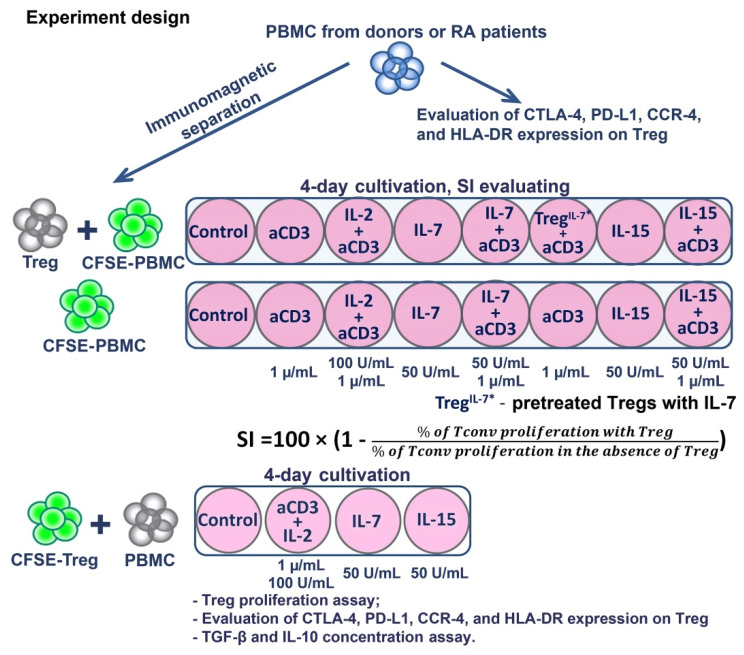
Experimental design.

To directly measure HP cytokines’ effects, CFSE-labeled Tregs with autologous PBMCs (30,000:30,000) were also cultivated with IL-7, IL-15, and anti-CD3 + IL-2 stimulation to assess their proliferation. The supernatants were sampled on Day 3 (according to the manufacturer’s instructions) to determine the concentrations of IL-10 (Vector-Best, Novosibirsk, Russia) and TGFβ (BioLegend, San Diego, CA, USA), the main Treg-suppressing cytokines, via ELISA. The expression of CTLA-4, PD-L1, CCR4, and HLA-DR on Tregs was estimated by flow cytometry before cultivation and on Day 4 of the experiment (Figure 1).

We decided to cultivate Tregs without prior activation to measure the initial Treg functionality and to bring the experiment as close to physiological conditions as possible. The optimal Treg/PBMC ratio was previously determined in experiments to be 1:1, based on existing research results on the topic [28,32,35]. Considering that the use of unirradiated PBMCs in a co-culture system has potential shortcomings related to the irrelevant activation of T cells or irrelevant cell death, we assessed these possible effects. We did not observe any significant cell damage in all the cultivation conditions or inappropriate proliferation in control wells without stimulation. However, it should be noted that the use of unirradiated rather than irradiated PBMCs was associated with an observed greater spread in the values of the suppression index and cell proliferation, due to the presence of other contaminating cells that can actively participate in the immune response in vitro.

### 2.3. Flow Cytometry

The phenotypes of Treg cells from peripheral blood were tested using the following BioLegend monoclonal antibodies: CD3 using FITC (or PE-Cy7 in the experiments to assess the suppressor index), CD4 using APC-Cy7, CD25 using PE, CD127 using PerCP-Cy5.5, CTLA-4 using APC, PD-L1 using APC, HLA-DR using APC, and CCR4 using PE-Cy7. Tregs were identified as CD4^+^CFSE^+^ when their phenotypic traits were assessed in the cultures (CD4 using APC-Cy7). Fluorescence minus one (FMO) controls were applied to the analyses, and a minimum of 5000 cells were acquired for each flow cytometric analysis, for which a BD FACS Canto II cytometer was used. All the previous steps (PBMC isolation, Treg sorting, cytometric analysis, and the initiation of cultivation) were performed on the day of blood collection without fixing or freezing the cells.

### 2.4. Statistical Analysis

The mean, median, standard deviation (SD), and interquartile range (IQR) were calculated. Shapiro–Wilk tests were used to test the normality of the data distributions. Unpaired Student’s *t*-tests or Mann–Whitney tests were used to compare the HCs and RA patients. ANOVA (the Freidman test in the case of nonparametric distributions) and post hoc analysis (Tukey’s and Dunn’s multiple comparison tests for parametric and nonparametric distributions, respectively) were used to compare multiple dependent groups. GraphPad Prism 7.03 was used to perform the statistical analysis.

## 3. Results

### 3.1. Strong T Cell Receptor (TCR) Signal in Combination with the Influence of Homeostatic Proliferation (HP) Cytokines Reduces Suppressive Activity of Tregs

**Figure 2:**
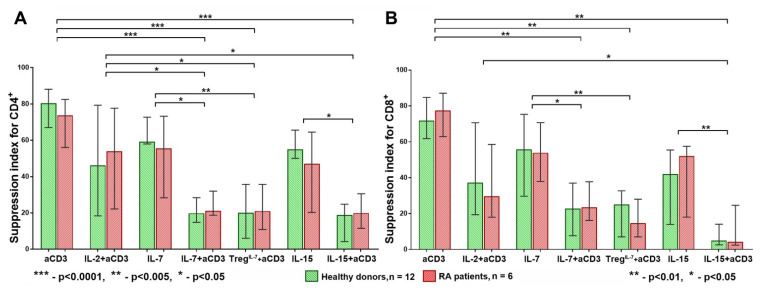
Treg suppression index for CD4^+^ (**A**) and CD8^+^ (**B**) cells. The suppression index (SI) was significantly reduced in both groups, healthy donors (HD) and rheumatoid arthritis (RA) patients (for both CD4^+^ and CD8^+^ cells), when the influence of homeostatic cytokines (IL-7 and IL-15) was accompanied by a strong T cell receptor (TCR) stimulation using anti-CD3 antibodies. There were no significant differences in SI between HDs and RA in all conditions of cultivation. Median and IQR. A comparison of related groups was performed using the Friedman test, post hoc analysis was performed using the Dunn test, and unrelated groups were compared using the Mann–Whitney test. RA, rheumatoid arthritis (n = 6); HDs, healthy donors (n = 12); SI, suppression index.

After four days of cultivation, we assessed Tregs’ suppressive activity under the conditions described above, finding that the anti-CD3, IL-7, IL-15, and anti-CD3 + IL-2 stimulations had similar effects on the Treg suppressive activity for the CD4^+^ cells in the healthy donors and RA patients. However, when we imitated the conditions of fast HP and exposed the cells to IL-7 or IL-15 accompanied by a strong [16] TCR stimulation (i.e., using anti-CD3 + IL-7 or anti-CD3 + IL-15), Tregs’ suppressive activity was significantly reduced, as the suppression index for CD4^+^ was significantly lower than that for the separate anti-CD3, anti-CD3 + IL2, IL-7, and IL-15 stimulations. A similar trend was observed when Tregs were pretreated with IL-7 (Figure 2A). Similar results were obtained for CD8^+^ cells (Figure 2B).

A high suppression index (SI) was observed when we applied IL-7 or IL-15 alone, anti-CD3, or anti-CD3 + IL2. By contrast, when IL-7 or IL-15 were combined with anti-CD3, or IL-7 was used for Treg pretreatment, the SI was significantly reduced (Figure 2B). A similar SI pattern was also observed for the CD4^+^ and CD8^+^ cells in the RA patients. The SI in the RA patients was significantly reduced when the cells were exposed to IL-7 or IL-15 accompanied by a strong TCR stimulation [16], with no significant differences revealed between the HDs and RA patients in all the cultivation conditions (Figure 2A,B). 

**Figure 3:**
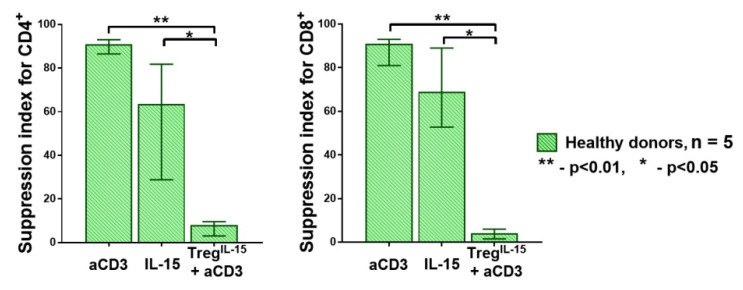
The SI for CD4^+^ and CD8^+^ cells in HDs (n = 5). The pretreatment with IL-15 had a direct effect on the Treg cells, reducing their suppressive function, similar to IL-7. Median and IQR. A comparison of related groups was performed using the Friedman test (ANOVA), and post hoc analysis was performed using the Dunn test. HDs, healthy donors; SI, suppression index.

It should be noted that the number of Treg cells was insufficient for testing the effect of IL-15 pretreatment on Treg function in a single experiment. Therefore, we performed an additional experiment where Treg cells from five healthy donors were pretreated with IL-15, similarly to the IL-7 pretreatment in the previous experiment. We revealed the same pattern observed for the IL-7 pretreatment. The Treg cells pretreated with IL-15 failed to suppress effector T cell proliferation (Figure 3).

**Figure 4:**
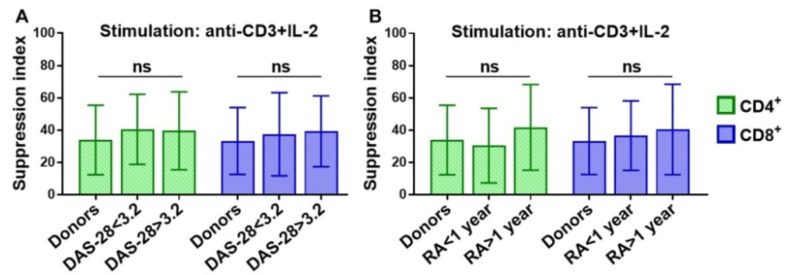
Treg suppression index for CD4^+^ and CD8^+^ cells in HDs and RA patients with different 28-joint disease activity score (DAS-28) results (**A**) and durations of disease (**B**). There were no significant differences in SI between HDs and RA patients with different DAS-28 results or disease durations for both CD4^+^ and CD8^+^ cells. Mean ± SD. Unrelated groups were compared using the Kruskal–Wallis test, and post hoc analysis was performed using the Dunn test. HDs, healthy donors (n = 18); RA, rheumatoid arthritis, DAS-28 < 3.2, n = 4; DAS-28 > 3.2, n = 9; duration < 1 year, n = 4; duration > 1 year, n = 9; SI, suppression index.

In order to find out whether disease activity or duration influenced the Treg suppressive activity, we additionally enrolled thirteen patients with different illness durations and DAS-28 results (Table 2) and assessed the suppressive activity of Tregs under anti-CD3 + IL-2 stimulation (Figure 4).

However, we did not reveal any significant differences in the suppression index of Treg cells between the HDs and RA patients with different disease durations and DAS-28 results. It is also worth noting that we did not find any significant correlations between the SI and clinical parameters such as the erythrocyte sedimentation rate (ESR) or C-reactive protein (CRP) (data not shown). Thus, the total pool of peripheral blood Treg cells in RA patients, independent of disease duration or activity, has the same suppression potential as the cells of healthy donors. 

**Figure 5:**
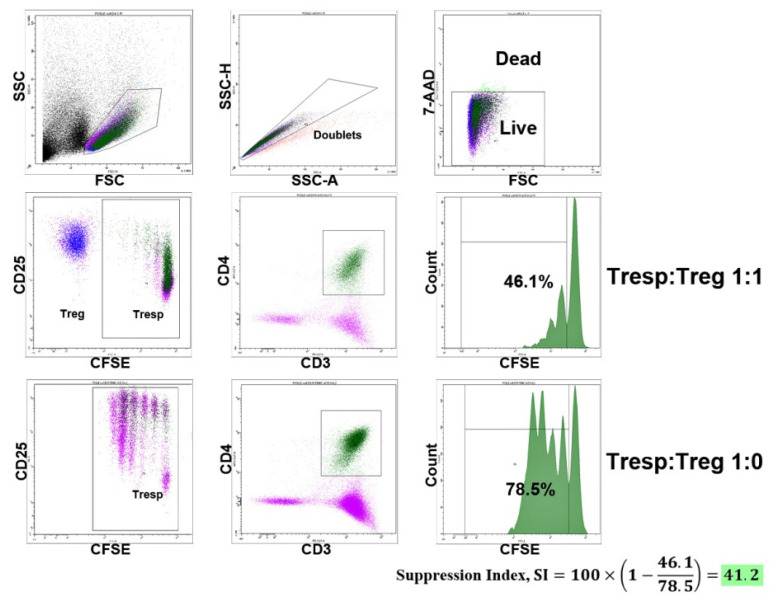
Example of the gating strategy for the evaluation of Treg SI for CD4^+^ cells. Treg cells were isolated using magnetic separation. For the suppression assay, PBMCs labeled with CFSE and Tregs were co-cultured in 1:1 ratio in different conditions of stimulation during 4 days. CFSE-labeled PBMCs were also cultivated under the same conditions without Treg cells to calculate the suppression index (SI). 7-AAD was used to live/dead discrimination.

The mechanism by which IL-7 or IL-15 in combination with anti-CD3 decreases the suppressive activity of Tregs is unknown, but it appears to be a mechanism that directly affects Treg cells, which can be confirmed by experiments when Tregs are pretreated with IL-7 or IL-15. Additionally, there are data showing that a strong TCR stimulation can lead to enhanced Granzyme B production by conventional T cells (Tconv) accompanied by an increased death of Tregs due to higher contact cytotoxicity [36]. Therefore, we analyzed Tregs’ suppressive activity and also evaluated the quantity of dead or damaged cells using 7-AAD, as described in a previous study (Figure 5) [37]. However, no decrease in Treg quantity under various stimulation conditions was observed (less than 0.5 ± 0.11% of dead Treg cells on average). Thus, the low SI is not associated with a decreased Treg number.

In addition, we evaluated the expression of HP cytokine receptors, CD127 and CD215, on CD4^+^ and CD8^+^ cells in the healthy donors and RA patients, finding no differences in the percentages or MFI values of CD127 and CD215 between the groups. We also observed that stimulation with HP cytokines (especially IL-7) significantly (*p* < 0.05) decreased CD127 expression and increased CD215 expression on the CD4^+^ and CD8^+^ lymphocytes in both groups. It should be noted that we did not find any differences in CD127 and CD215 (MFI) expression on Treg cells between the healthy donors and RA patients before and after stimulation (data not shown). 

**Figure 6:**
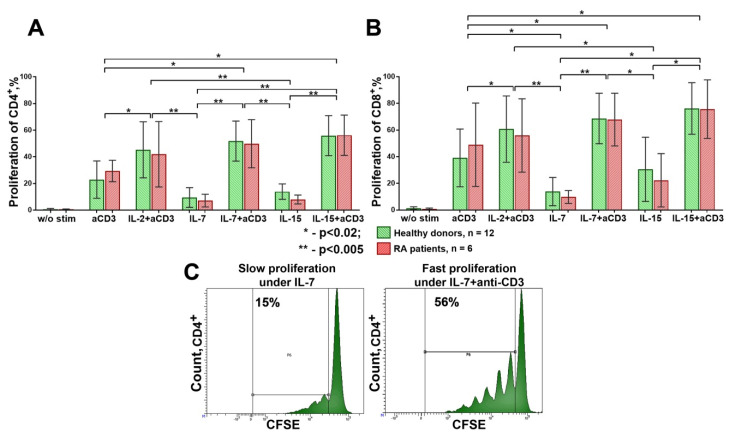
Proliferation of CD4^+^ (**A**) and CD8^+^ (**B**) cells in healthy donors (n = 12) and RA patients (n = 6); (**C**) example of slow and fast proliferation (for an example of one of the donors). Significantly higher proliferation of CD4^+^ and CD8^+^ cells was observed when the influence of cytokines (IL-2, IL-7, or IL-15) was accompanied by TCR stimulation with anti-CD3 antibodies. Mean ± SD. A comparison of related groups was performed using one-way analysis of variance for dependent groups (RM one-way ANOVA), and post hoc analysis was performed using Tukey’s tests. Unrelated groups were compared using unpaired Student’s *t*-tests. RA, rheumatoid arthritis.

It is worth noting that the direct influence of stimulation factors on Tconv could affect the ability of Tregs to inhibit Tconv proliferation. Therefore, the proliferative activity of CD4^+^ and CD8^+^ cells significantly varied in different cultivation conditions. As expected, the highest proliferation rate was observed when anti-CD3 was combined with IL-2, IL-7, or IL-15. At the same time, a low proliferation rate was observed when cells were cultivated with IL-7 or IL-15 alone (Figure 6). Such a low proliferation rate is assumed to be an approximation of slow HP, while the high proliferation caused by a strong TCR stimulation [16] with HP cytokines (anti-CD3 + IL-7 or anti-CD3 + IL-15) is likely to imitate fast HP. It should be noted that no significant differences were found in the proliferation of Tconv between the donors and RA patients under all the cultivation conditions (Figure 6). Despite the high proliferation rate of the CD4^+^ and CD8^+^ cells stimulated by anti-CD3 + IL-2, the SI was also high in both the HDs and RA patients. This was not the case for the anti-CD3 + IL-7 and anti-CD3 + IL-15 stimulation, indicating that IL-7 and IL-15 are not able to replace IL-2 and cannot effectively support the functional activity of Tregs in conditions close to a strong TCR stimulation.

Thus, the results obtained indicate that the simultaneous action of HP cytokines and a strong TCR signal [16] impairs Tregs’ capacity to suppress Tconv proliferation.

### 3.2. The Influence of HP Cytokines on Proliferation and Expression of Functional Molecules of Treg Cells

Considering the direct effect of the HP cytokines on Treg cells’ suppressive activity found in previous experiments, in a further step, we investigated the proliferation and phenotypical changes of Treg cells under the direct influence of IL-7 and IL-15.

**Figure 7:**
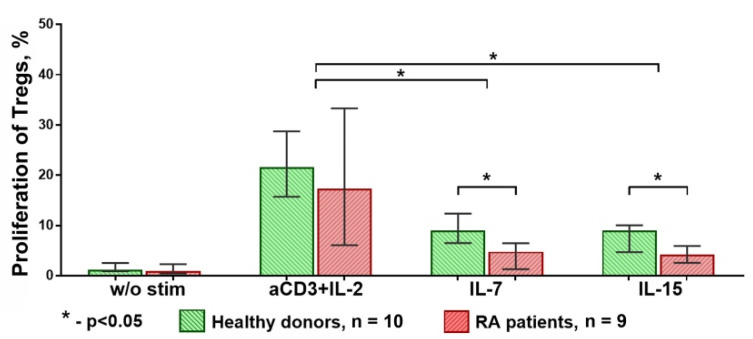
Proliferation of Treg cells in HDs and RA patients. Proliferation of Treg cells from RA patients under IL-7 or IL-15 stimulation was significantly lower than proliferation of those from HDs under the same. Median and IQR. Unrelated groups were compared using Mann–Whitney tests. A comparison of related groups was performed using the Friedman test, and post hoc analysis—the Dunn test. RA, rheumatoid arthritis (n = 9); HDs, healthy donors (n = 10).

The highest Treg proliferation was observed when the cells were stimulated by anti-CD3 + IL-2, with the results being similar in the HD and RA groups. However, the proliferation of Treg cells under IL-7 or IL-15 stimulation was significantly decreased in the RA group (Figure 7).

The nature of this phenomenon is unknown, and additional research is required. It should be noted that immunosuppressive therapy could make a significant contribution to decreasing the proliferative activity of Tregs in RA patients; nonetheless, we did not reveal any differences in the proliferation of CD4^+^ or CD8^+^ cells between the HDs and RA patients under any of the stimulation conditions (Figure 6).

In the next stage of the study, we explored the effect of HP cytokines on the expression of functional molecules of Tregs in vitro. The influence of different stimulation conditions (without stimulation, anti-CD3 + IL-2, IL-7, and IL-15) was investigated using purified, CFSE-labeled CD3^+^CD4^+^CD25^+^Cd127^lo^ Treg cells cultivated with autologous PBMCs at a 1:1 ratio.

**Figure 8:**
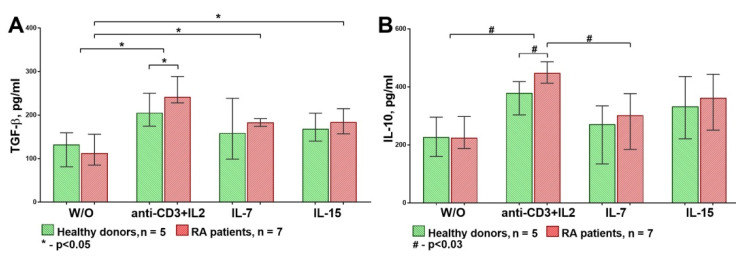
TGF-β (**A**) and IL-10 (**B**) concentrations in supernatants of Treg/peripheral blood mononuclear cell (PBMC) cultures. Production of TGF-β and IL-10 caused by anti-CD3 + IL-2 stimulation was significantly higher than under IL-7 or IL-15 influence in both groups. In addition, the production of these cytokines was significantly higher in RA patients than in HDs under anti-CD3 + IL-2. Median and IQR. Unrelated groups were compared using the Mann–Whitney test. A comparison of related groups was performed using the Friedman test (ANOVA), and post hoc analysis was performed using the Dunn test. RA, rheumatoid arthritis (n = 7); HDs, healthy donors (n = 5); W/O, without stimulation.

First, we assessed the TGF-β and IL-10 concentrations in supernatants on the third day by ELISA (Figure 8). 

The statistical analysis revealed that the anti-CD3 + IL-2 stimulation significantly increased TGF-β and IL-10 production in both groups compared to the control levels when no stimulation was applied. In this study, IL-7 and IL-15 significantly increased TGF-β production only in the RA group. Moreover, TGF-β and IL-10 production was significantly higher in the RA patients than the healthy donors under the anti-CD3 + IL-2 stimulation.

**Figure 9:**
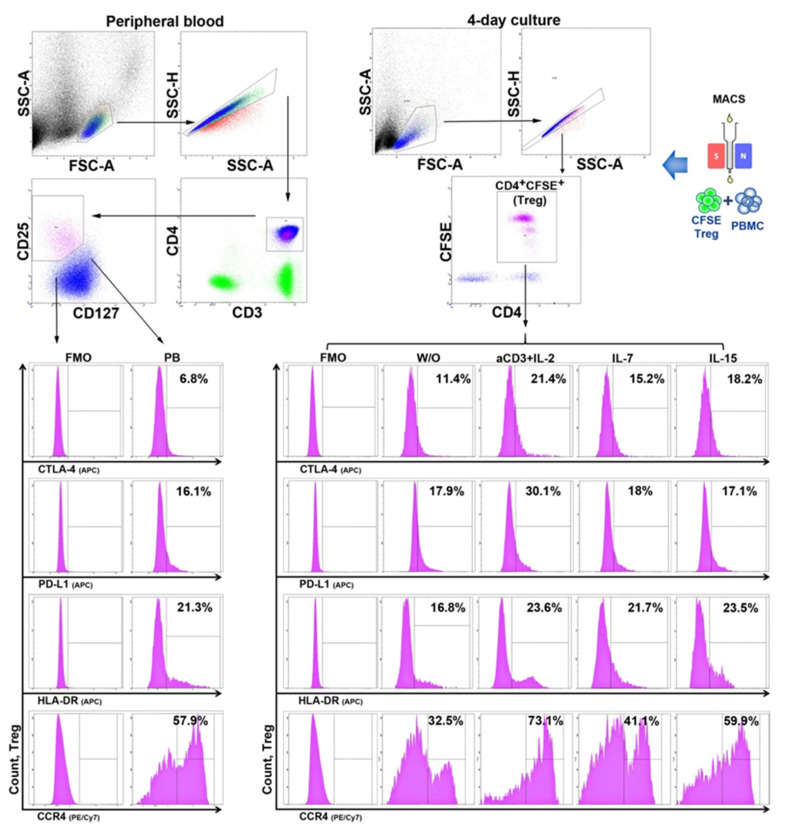
Example of gating strategy for CTLA-4, PD-L1, HLA-DR, and CCR4 expression on Treg cells from peripheral blood and after cultivation (for an example of one of the RA patients). FMO, fluorescence minus one; W/O, without stimulation.

In addition to the evaluation of the TGF-β and IL-10 concentrations in supernatants, we also assessed the extracellular expression of PD-L1, CTLA-4, HLA-DR, and CCR4 on Treg cells. The gating strategy is shown in Figure 9.

Thus, when anti-CD3 + IL-2 stimulation was used, the Treg cells of the RA patients showed a higher ability to produce anti-inflammatory cytokines and were characterized by greater CCR4 expression than those from the HDs, which may indicate their activated status in patients with RA. These data are consistent with the cytometric analysis of Treg cells from peripheral blood conducted before cultivation, and revealed a higher frequency of CCR4^+^Tregs in peripheral blood accompanied by a lower expression of CTLA-4. Thus, Treg cells from RA patients demonstrate a suppressive phenotype and exhibit a greater potency to produce anti-inflammatory cytokines in vitro under anti-CD3 + IL-2 exposure.

**Figure 10:**
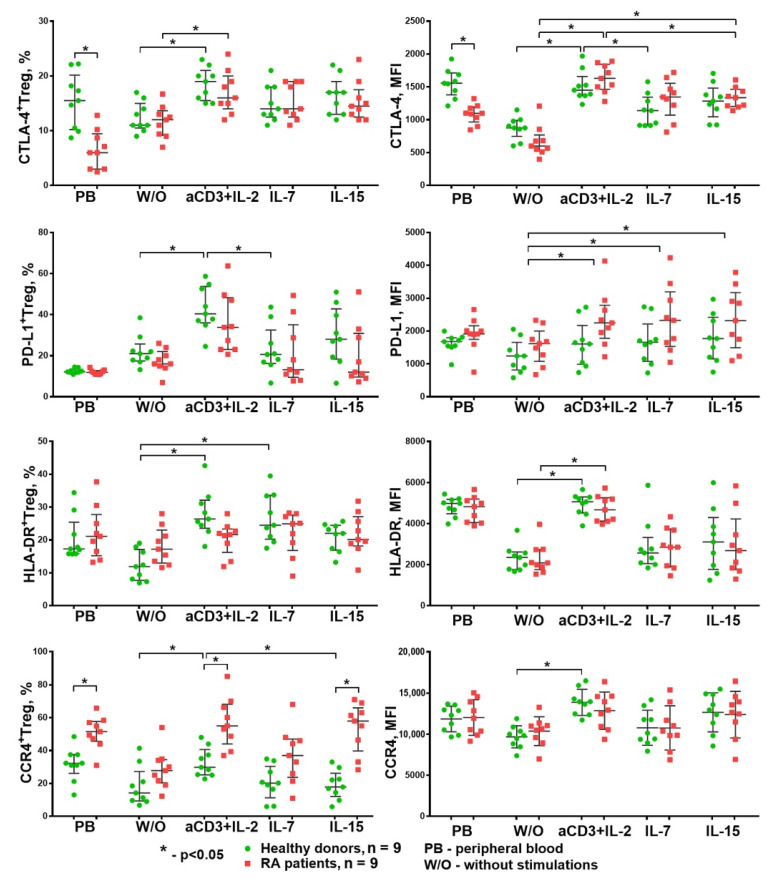
Expression of different functional molecules on Treg cells during cultivation. Anti-CD3 + IL-2 stimulation increased the expression of assessed functional molecules of Treg cells in both HDs and RA patients in a similar manner. By contrast, stimulation with homeostatic cytokines increased only the density of PD-L1 expression in both groups. Median and IQR. Unrelated groups were compared using the Mann–Whitney test. A comparison of related groups was performed using the Friedman test (ANOVA), and post hoc analysis was performed using the Dunn test; RA, rheumatoid arthritis (n = 9); HDs, healthy donors (n = 9).

The statistical analysis revealed the following changes (Figure 10): anti-CD3 + IL-2 stimulation significantly increased the percentage of Treg cells expressing CTLA-4, PD-L1, HLA-DR, and CCR4 in the healthy donors. The expression density (MFI) of these functional molecules on the Tregs’ surfaces also increased. At the same time, HP cytokines only significantly increased the expression density of PD-L1 (MFI) on Treg cells. Similar results were also obtained for the RA patients. It should be noted that the Treg cells from the RA patients differed from the healthy donors’ by their higher CCR4^+^ Treg percentage, both in the peripheral blood and under the influence of anti-CD3 + IL-2 and IL-15. Thus, the only difference between the HD and RA groups was the expression of the CCR4 chemokine receptor (a receptor for the chemokines CCL22 and CCL17), which facilitates migration to sites of inflammation.

We also analyzed the phenotype of proliferating CFSE^lo^Treg cells and compared it to that of nonproliferating CFSE^hi^Treg cells. We revealed the following features: the CFSE^lo^Treg cells had higher CD25 (MFI) and HLA-DR (% and MFI) expression than the nonproliferating Treg cells and had a similar expression of CD127, CTLA-4, and CCR4 (% and MFI). In addition, the CFSE^lo^Treg cells had a slightly lower expression of PD-L1 (% and MFI). Such differences were observed for all the stimulation conditions in both the HD and RA groups (data not shown).

## 4. Discussion

This study pursued two main objectives: to compare the suppressor activity of Tregs between healthy donors and patients with rheumatoid arthritis and to assess the effect of humoral factors of homeostatic proliferation on the suppressive activity of Treg lymphocytes.

It was not possible to detect any difference in the Tregs’ suppressive activity between the healthy donors and RA patients in the experiments, which corresponds to previously published data [30,32,38]. This can be regarded as evidence that the suppressive potential of the total Treg pool in patients with RA is preserved at the level of that in healthy donors with the absence of Treg intrinsic defects. This has been confirmed by studies that revealed no differences between healthy donors and RA patients in terms of gene expression profiles and the comprehensive analysis of miRNA expression in Treg cells from peripheral blood [32,39]. Therefore, the resistance of responder cells to suppression may explain the impaired Treg suppression of cell proliferation and cytokine production by effector cells from the site of inflammation in RA. It should be noted that Treg cells obtained from the peripheral circulation may not accurately reflect Treg function at the site of inflammation because the proinflammatory milieu may reduce the functional activity of Tregs and cause the resistance of Tconv to Treg-mediated suppression [40]. Thus, elevated levels of IL-7 and IL-15 in synovia [41,42], along with elevated levels of inflammatory cytokines, may have additional negative effects on the functional activity of Treg cells in rheumatoid arthritis. Additionally, it should be noted that the usage of glucocorticoids in the treatment of rheumatoid arthritis can potentially restore the functional activity of T-regulatory cells [43]. Therefore, studies of the suppressive function of T-regulatory cells at the earliest stage of rheumatoid arthritis, ideally before starting therapy, can more accurately reflect the state of the T-regulatory branch of immunity.

HP cytokines, IL-7 and IL-15, applied separately, and thus simulating slow proliferation conditions, had no influence on the Tregs’ suppressive activity. However, a combination of IL-7 or IL-15 with anti-CD3 significantly decreased the ability of Tregs to suppress the proliferation of conventional T cells, both CD4^+^ and CD8^+^. As noted above, such stimulation is similar to the conditions of fast homeostatic proliferation in vivo when deep lymphopenia is observed with a large number of free niches simultaneously present. The decrease in the number of producer cells due to lymphopenia results in a relative deficiency of IL-2, which may be an additional factor of Tregs’ decreased functional activity in vivo. This may lead to the proliferation of various T cell clones, including self-reactive clones [18,19,44]. As noted earlier, this is due to the competition between different T lymphocyte clones for proliferation signals, with the signal transmitted via TCRs playing the most important role; in this competition, the advantage belongs to those lymphocyte clones whose TCRs have a higher affinity to bind the peptide in the MHC complex. Given a limited number of foreign antigens presented in the body simultaneously and a continuous cross-presentation process, the majority of the presented antigens are epitopes of self-proteins. That is why it can be assumed that the majority of cells that enter homeostatic proliferation are Tconv with a higher TCR affinity for self-peptides along with a small number of clones specific to foreign antigens that are currently present in the body. Thus, as a result of rapid homeostatic proliferation, self-reactive clones of T cells are selected to proliferate. This leads to a decrease in TCR diversity and increases the risk of autoimmunity. Although the difference between slow and fast HP is, to some extent, arbitrary, fast HP may lead to qualitative changes in the immune system. If slow HP is considered to be an analog of physiological regeneration, then fast HP may be identified with emergency tissue regeneration damaging its structure and functions [15]. Thus, we revealed the inability of Tregs to suppress the fast proliferation of Tconv cells caused by a strong TCR signal and HP cytokines, which may represent an additional mechanism linking homeostatic proliferation and autoimmunity.

The ability of Tregs to suppress the proliferation of Tconv in response to a strong stimulation of TCR [16] was also reduced by the pretreatment of the Tregs with IL-7 or IL-15, indicating their direct negative influence on the Tregs’ suppressive function. This was consistent with previously obtained data [45,46], which showed a decrease in the expression of *EOMES* and *NF-κB* and an increase in the expression of *IL-6* and *INF-γ* in Treg cells under the influence of IL-7. However, there is still not enough reliable evidence to connect the change in the expression of these genes with a decrease in Tregs’ suppressive activity, which establishes the groundwork for future research.

Nonetheless, we can hypothesize that the stimulation of T cells by HP cytokines (IL-7 or IL-15) in combination with a strong TCR signal (from anti-CD3 antibodies) may lead to the hyperactivation of the PI3K–Akt pathway due to a cumulative effect and, thus, cause the resistance of Tconv cells to Treg-mediated suppression [12,13,16,40]. A similar effect was demonstrated for Tconv cells in a previous study, where TNFα and IL-6 induced PKB/c-Akt activation and, thus, provided resistance to Treg-mediated suppression [40].

For a more detailed study of the HP cytokine effect on Treg suppressor activity, we evaluated the expression of several functional molecules on the Treg surface. It is well known that one of the important Treg suppressor molecules is CTLA-4. It binds to the CD80/86 co-stimulation molecules on dendritic cells and removes B7 complexes from their surface through trans-endocytosis [47]. Another molecule mediating the suppressor functions of Tregs is PD-L1; it interacts with PD-1 on activated effector cells and causes their anergy and apoptosis or even induces Tregs de novo [48]. The chemokine receptor CCR4 is the only receptor for the chemokines CCL22 and CCL17. Both ligands are known to evoke the chemotaxis of CCR4-bearing Treg cells to draining lymph nodes and to mature dendritic cells expressing CCL17 and CCL22, which is critical for Treg cells’ suppressive function in vivo [49,50]. In addition, CCR4^+^ Treg cells are characterized by an activated phenotype and high suppressive activity [51,52]. HLA-DR is one of the key cell surface molecules expressed on antigen-presenting cells, but is also expressed on activated Treg cells with high FoxP3 expression [29,53]. Such Tregs with HLA-DR expression exhibit early contact suppression activity [54]. The analysis of the influence of HP humoral factors on the expression of the functional molecules by Treg lymphocytes showed that the greatest suppressor potential that Tregs acquired was under the influence of the combination anti-CD3 + IL-2. It significantly increased the percentage of Tregs expressing PD-L1, CTLA-4, HLA-DR, and CCR4, as well as the membrane expression density (MFI) of these molecules, while IL-7 and IL-15 only increased the expression density (MFI) of PD-L1. The observed increase in the percentage of PD-L1^+^ and CTLA-4^+^ Tregs and the expression density of PD-L1 and CTLA-4 (MFI) may indicate an increase in the suppressor potential of Tregs under anti-CD3 + IL-2 stimulation. The revealed pattern also corresponds to the published data on the effect of cytokines with a common γ-chain on PD-L1 expression by CD4^+^ lymphocytes [55]. However, given that IL-7 and IL-15 could not increase the number of PD-L1^+^ and CTLA-4^+^ Tregs, it may be concluded that HP cytokines cannot provide the same level of Treg suppressive activity as anti-CD3 + IL-2. Given that CCR4 can also be used as a potential marker of effector Treg lymphocytes [51], the increased expression of this molecule together with CTLA-4, PD-L1, and HLA-DR indicates the ability of the anti-CD3 + IL-2 stimulation to cause Tregs’ activation and transition into effector Treg cells. This suggestion is indirectly confirmed by the higher concentrations of TGF-β and IL-10 in the supernatants following anti-CD3 + IL-2 stimulation.

Importantly, there was no difference in the expression of all the analyzed molecules between the groups of donors and patients, except for CCR4, which was significantly higher in the peripheral blood of the RA patients and when anti-CD3 + IL-2 and IL-15 stimulation was applied. Similar results were obtained for IL-10 and TGF-β production. The anti-CD3 + IL-2 stimulation significantly increased their concentrations in the supernatants in both the HD and RA groups. The concentrations of these cytokines were also significantly higher in the RA group than the HDs with this type of stimulation. Thus, the RA group showed an increased percentage of CCR4^+^ Tregs and a higher production of IL-10 and TGF-β, which may indicate a compensatory activated state of Treg cells in rheumatoid arthritis that could be caused by physiological mechanisms or the medications applied for treatment. Therefore, the persistence of inflammation in synovia in rheumatoid arthritis is likely to be caused by the resistance of T responders to Treg suppressive signals [35,38,40].

## 5. Conclusions

In this study, we identified a new potential mechanism of autoimmunity development: Treg cells fail to suppress the fast homeostatic proliferation of T lymphocytes. We have shown that Treg cells cannot effectively suppress the proliferation of T lymphocytes that receive a strong TCR signal under the influence of the homeostatic cytokines IL-7 and IL-15.

We have also shown the preserved functional activity of peripheral Treg cells in rheumatoid arthritis. However, this does not exclude the presence of any other defects at the clonal level. Thus, further investigations at the single-cell level are required.

## Figures and Tables

**Table 1:** Evaluated clinical parameters. DAS, disease activity score; ESR, erythrocyte sedimentation rate; CRP, C-reactive protein; TJC, tender joint count; SJC, swollen joint count; SD, standard deviation.

Parameter	Healthy Donors	RA Patients
Male	6	3
Female	12	11
Age, mean ± SD	56.4 ± 11	59.5 ± 11
DAS-28, mean ± SD	-	5.2 ± 1.6
ESR (mm/h), mean ± SD	-	35.4 ± 9.4
CRP (mg/dl), mean ± SD	-	19.9 ± 6.7
TJC, mean ± SD, out of 28	-	7.3 ± 3.2
SJC, mean ± SD, out of 28	-	4.2 ± 2.9
Duration of disease (y)	-	5.5 ± 2.7

**Table 2:** Evaluated clinical parameters. DAS, disease activity score; SD, standard deviation; RA, rheumatoid arthritis; HDs, healthy donors; N, sample size.

Groups	N	Age (Mean ± SD)	Male	Female	Duration (Mean ± SD)	DAS-28 (Mean ± SD)
DAS-28 < 3.2	4	62.5 ± 18.9	-	4	15 ± 2.1 years	2.01 ± 0.82
DAS-28 > 3.2	9	59.1 ± 9.1	1	8	6 ± 4.6 years	5.58 ± 0.99
RA < 1 year	4	60 ± 4.5	-	4	9 ± 2 months	5.92 ± 1.61
RA > 1 year	9	59.7 ± 13.2	1	8	10.1 ± 4.8 years	4.10 ± 1.78
HD	18	56.4 ± 11	6	12	-	-

## Data Availability

The datasets used and/or analyzed during the current study are available from the corresponding author on reasonable request.
